# Flavine: the Ideal Antiseptic

**Published:** 1917-03-24

**Authors:** 


### FLAVINE.

### The Ideal Antiseptic.

The sudden enormous increase in septic wounds of all kinds which marked the commencement of hostilities two and a half years ago, quickly caused the medical profession to recognise that nearly all the antiseptics in common use were grossly defective as agents to cleanse and promote the speedy healing of severely infected lesions. This, it is true, had been in some degree realised in the transition from "antiseptic" to "aseptic" surgery. Strong antiseptic agents were known to be actively harmful to the tissues themselves, and hindered rather than aided the process of healing.

But since in civilian surgery septic wounds are the exception, and possibly also owing to the fact that our last great campaign, the Boer War (which marked the introduction of iodine), was remarkable for the number of wounds which healed by first intention, the problem of finding an efficient antiseptic had been strangely overlooked, and surgeons were content to confine themselves to two or three to which they were accustomed and of whose bactericidal properties there was no question.

### Theoretical Considerations.

It is obvious, however, that an ideal antiseptic should possess certain properties very different from those of iodine or corrosive sublimate. These act as general protoplasmic poisons, having no specific action whatever upon bacteria but behave, so to speak, as an explosive whose violence it is impossible to direct. They destroy all life equally; kill off the phagocytic cells in the blood, healthy tissue cells, and are, in fact, in most cases more toxic to the body than to the bacteria invading it. Further, antiseptics in general are "fixed" by protein—that is, they enter into some sort of combination with the blood serum, so that their efficiency in this medium is far less than in watery solution. Corrosive sublimate, for example, has an efficiency one hundred times greater in an aqueous than in a serum medium. Moreover, they tend to leave a crust of dead material upon a wound, beneath which organisms are free to multiply, so that the last state of the patient is often worse than the first.

Theoretically, therefore, an antiseptic should embody some or all of the following properties.

1. It should be strongly bactericidal in the presence of protein material—*i.e.* blood serum.
2. It should have no effect on phagocytosis when employed in concentrations sufficient to kill micro-organisms.
3. It should be capable of application to delicate membranes, *e.g.* the conjunctiva.
4. It should act as a stimulant to healthy tissue, thus promoting the natural process of repair.
5. If absorbed into the system it must not be toxic for any specialised tissue or exert any deleterious influence upon metabolism.

Since the general acceptance of these principles many attempts have been made to substitute for the old-fashioned blunderbuss methods, involving wholesale destruction of the very cells whose existence is essential to any process of repair, an exact weapon which would destroy the invading organisms, and at the same time leave the body free to organise its own defences.

As Sir Almroth Wright pointed out, the serum poured out on an inflamed surface contains, in addition to an abundance of white blood corpuscles, the ferment antitrypsin, which serves in some way to stem the invading tide of microbes. Now, the warrior cells contain in their bodies the ferment trypsin, which serves to dissolve the bacteria which they ingest; upon their death, however, it is liberated into the surrounding serum and neutralises a certain amount of the antitrypsin, thus actually facilitating the progress of the hostile bacteria. Wright, therefore, conceived the idea that a hypertonic solution of saline (containing a small amount of citrate to retard clotting) would, if applied continuously to a freely exposed wound, greatly hasten healing by purely natural means. A lavish outpouring of serum would be furnished by the tissues to dilute the saline down to the normal, and this in its turn would call out an army of leucocytes, whilst the toxins of the bacteria would be removed by the constant flow of solution. This is known as the physiological method of wound treatment, and it has been employed extensively in base hospitals both here and in France. But although this was undoubtedly a step in the right direction, the ideal antiseptic was yet to be found. Experiments were conducted on a large scale with a solution introduced by Dr. Dakin, known as Dakin's solution, and subsequently modified by Daufresne and others. This consisted of 1.4 per cent. carbonate of soda, to which were added 2 per cent. chloride of lime and .4 per cent. boric acid. Its efficacy lay mainly in the chlorine ions liberated.

### An Experimental Hospital.

The later modification contained no boric acid and a smaller percentage of hypochloride. Using this solution, Dr. Carrel, a French-American surgeon, has achieved some striking results in the hospital equipped by him at Compiègne. How far, however, this is a result of his novel and brilliant technique (and the minute care devoted to each case) it is not easy to say. The ambulance has been equipped frankly as an experimental station, the surroundings are almost luxurious, and the medical staff (seven qualified men to eighty beds) and the number of nurses and orderlies far in excess of an ordinary base hospital. Less striking though still remarkable results have been obtained under routine conditions by Dr. Chutro, at the Lycée Pasteur in Paris, by following Carrel's methods, with modifications towards simplicity. The system consists, briefly, of the irrigation of wounds which have been previously opened surgically by means of special apparatus, so devised that a small quantity of the solution, diluted to 0.45 to 0.50 per cent., can be let in every two hours without disturbing the dressings covering the wound. These are removed, in the case of the Lycée Pasteur, once every two days, and minute care is taken to avoid handling any tube or dressing going into the wound. The solution has been found to be non-irritating, capable of dissolving away quantities of necrosed tissue, and further neutralising the toxic products of bacterial activity. Remarkable results are reported from its use, and grossly contaminated wounds, after periods of treatment varying from five to twenty-five days, were shown to be free from bacteria, and could be stitched up. A large proportion so closed healed by first intention, and of the small percentage which showed signs of inflammation two only had to be reopened for further treatment.

But important as these results have been, it is evident that Dakin's solution fulfils only part of the conditions laid down for the ideal antiseptic, since it is equally toxic for the white cells in the blood stream and has no stimulating action on the process of tissue repair. Research, therefore, sought some drug which, whilst destructive of the bacteria usually found in septic lesions, the staphylococcus, streptococcus, *B. coli*, etc., should have no inhibitory action on the germ-destroying power of the white cells. The possibility of such a drug had already been demonstrated by the discovery of salvarsan, which was highly toxic to spirochætes but harmless to the blood-cells. Later work by Ehrlich upon the trypanosomes, the parasite causing sleeping-sickness, had shown that certain members of the chemical group known as the acridines, substances closely allied to the aniline dyes of commerce, were poisonous to these organisms, whilst certain of them, crystal violet, brilliant green, and flavine, had no appreciable effect on normal body tissue.

By great good fortune Dr. Browning, the present director of the Bland-Sutton Institute of Pathology at the Middlesex Hospital, had chanced to be one of Ehrlich's co-workers in these investigations, and on his return to England brought with him a small quantity of the substance flavine. Here for some time the matter rested.

But in the course of a series of experiments on antiseptics, comprising those in common use and certain others, such as malachite green, which are used but rarely, this work of Ehrlich's was called to mind. The result was the discovery of the simple but hitherto unsuspected fact *that germicidal potency and inhibition of phagocytosis do not necessarily run parallel.*

In other words, whilst the common antiseptics all interfered seriously with the work of the white cells at concentrations insufficient to kill the bacteria usually found in septic wounds, certain of the acridine series were lethal to bacteria in concentrations far less than that required for bacterial destruction. To express the relation of these two factors a figure is used known as the therapeutic coefficient.

This represents the numerical value of the fraction—

$$\frac{\text{Concentration which reduces phagocytosis by 50 per cent.}}{\text{Lethal concentration in serum,}}$$

where the lethal concentration is that which just suffices to kill micro-organisms, as tested by sub-culture. Thus, for example, in the case of phenol, a solution of 1 in 250 suffices to kill cocci, whilst the phagocyte count is reduced to 50 per cent. of control by a solution of 1 in 500. The therapeutic coefficient is therefore $\frac{.02}{.04} = 0.5$. In the case of the *bacillus coli communis*, the two concentrations are exactly the same and the therapeutic coefficient is 1. ($\frac{.02}{.02} = 1.$)

It is obvious, then, that the higher the therapeutic coefficient, the nearer from one standpoint does the antiseptic approach to the ideal conditions laid down at the beginning of this article.

The various values for T.C. in these experiments are not without interest and may be briefly summarised in tabular form:—

| Antiseptics | T.C. for cocci | T.C. for *B. coli communis* |
|---|---|---|
| Eusol (0.34 available cl.) ... | 0.25 | 0.25 |
| Dakin's solution (modified) ... | 0.25 | 0.25 |
| Carbolic acid ... ... ... | 0.5 | 1 |
| Corrosive sublimate ... ... | 1.4 | 1.4 |
| Iodine (in K.I.) ... ... | 0.2 | 0.2 |
| Brilliant green sulphate ... | 15 | 1.7 |
| Brilliant green oxalate ... | 14 | 0.5 |
| Malachite green ... ... ... | 6 | 0.15 |
| Crystal violet ... ... ... | 57 | 1.1 |
| Flavine ... ... ... ... | 400 | 200 |

Flavine was thus shown to be incomparably the most powerful of all the substances experimented on, since a solution of 1 in 100,000 was lethal to *B. coli*, half that strength sufficed to kill cocci, whilst phagocytosis was not inhibited till 1 in 500 had been reached. These observations have furthermore been borne out by the results of extensive clinical investigations in the various departments of the Middlesex Hospital, where it has been employed for the last fourteen months. In the outpatient department between two and three thousand cases have been treated with flavine and brilliant green, approximately half with each substance; the cases have been taken indiscriminately, there has been no selection. The cases treated fall into two main categories: (1) those in which suppuration had already occurred, and (2) those in which the patient presented himself for treatment a few hours after the injury had been inflicted.

The former were treated by free incision, swabbing with 1:1,000 malachite green or flavine in normal saline, and the injured area was then covered with gauze soaked in the solution and covered with protective padding to prevent evaporation. Swabbing or syringing of the wound has been carried out once or twice daily, according to the acuteness of the condition.

"Between two and three hundred cases of septic hand and finger affections"—to quote from the report—"have been so treated, and on the average marked improvement as regards both reduction of inflammatory swelling and discharge of pus has been evident in two days; even in the most severe cases healing has been complete in fourteen days. Results of treatment of large superficial infected wounds have been highly satisfactory, the noteworthy points being the very rapid formation of healthy granulations and also the great rapidity with which the epidermis covered the area."

In the cases treated before suppuration had occurred the vast majority, when promptly treated with these solutions, can be at once closed and will heal by first intention, however heavily they may be contaminated by dirt, hair, clothing, etc. In more severe cases, such as compound fractures, similarly treated, healing has been equally remarkable, the rapidity with which *sequestra* come away being a notable feature.

Flavine is apparently non-irritant to delicate surfaces such as the conjunctiva in 1:1,000 concentration, and as much as 300 c.c. have been injected intravenously without ill effect.

A few c.c. are commonly injected into the subcutaneous tissue around a wound for prophylactic purposes. It is excreted unchanged in the urine, and should therefore act as a urinary antiseptic.

The difficulty at the present time consists in obtaining sufficient quantities of the drug to profit by its exceptional qualities at a time when the need for them is probably greater than ever before. Owing to the disorganised state of the aniline-dye industry, all acridine compounds are scarce, and the synthesis of flavine is one only to be undertaken by highly skilled chemists. The Lister Institute is at present engaged in its production, and when a satisfactory technique has been worked out its manufacture on a commercial scale will be considered. The question is certainly one of great urgency. Apart from the immense reduction of suffering to our wounded, the saving to the country which would be effected by restoring an injured man to the fighting line or the ranks of industry in a quarter the time now taken cannot be overestimated. It is, indeed, a marvel to anyone familiar with the liberal encouragement afforded by other nations to scientific research to find that work of this kind should in England be so often left to the enterprise and self-devotion of a few individuals, aided, perhaps, by grants from private societies, giving to the cause their none too ample leisure and receiving far from adequate payment. Like so many of the triumphs of modern research, the discovery of flavine has not been the result of a sudden happy inspiration. The road to success has been long and arduous, the triumphant "eurekas" few and far between. "*Dans les champs de l'observation,*" said Pascal in a famous dictum, "*le hasard ne favorise que les esprits préparés.*" To Dr. Browning and his loyal band of co-workers the admiration and humble thanks of the nation are due. Let the authorities see to it that their labour has not been in vain, and that the preparation of flavine on a large scale is undertaken with as little delay as possible.

